# Supplementary material for: Integrated Analysis of Gene Expression Differences in Twins Discordant for Disease and Binary Phenotypes
Source: Sci Rep. 2018 Jan 8;8:17. doi: 10.1038/s41598-017-18585-3 (PMC5758574; doi:10.1038/s41598-017-18585-3)

Supplementary Document

Integrated Analysis of Gene Expression Differences in Twins Discordant for Disease and Binary Phenotypes

Sivateja Tangirala and Chirag J Patel

Table S1. Overlapping significantly expressed genes in discordant twins. Raw number of overlapping genes that were called significant (FDR < 0.05 and absolute value of mean difference greater than 0.95 percentile) genes for pairs of phenotypes.

Table S2. Overlapping number of measured genes. Pairwise overlapping number of measured genes for pairs of phenotypes.

Table S3. Pre- and post-COCONUT correlation of mean differences for FDR significant genes (FDR < 0.05 and overlapping on all experiments). Correlations of the mean differences of FDR significant genes (measured in all 7 studies) pre- and post-COCONUT normalization.

Table S4. Pre- and post-COCONUT correlation of mean differences for overlapping 16,256 genes. Correlations of the mean differences of genes measured in all 7 studies (total of 16,256 genes) pre- and post-COCONUT normalization.

Table S5: Absolute Effect Size thresholds for each Phenotype. This table includes the absolute effect size threshold (of the 95th percentile of mean differences) for each phenotype and for the meta-analysis.

Figure S1. Phenotype-specific cumulative distribution function (CDF) of gene expression differences. The cumulative distribution function of mean expression differences for each of the 7 phenotypes.

Figure S2. Meta-analytic Cumulative Distribution Function (CDF) of gene expression differences across all phenotypes. The cumulative distribution function of mean expression differences for the meta-analytic effect sizes (mean expression differences).

Figure S3. I^2 values versus significance of I^2 (FDR-corrected QEp) values. I^2 (heterogeneity estimate) values versus the negative log (base 10) of the FDR-corrected QEp values for all the measured genes that were obtained from the meta-analysis of mean-differences for each gene across all seven phenotypes.

Table S1. Overlapping significantly expressed genes in discordant twins.

Phenotype	PA	UC	IAR_invitro	CFS	IQ	MDD	OB
PA	15	1	0	0	0	0	0
UC	1	424	2	0	16	1	13
IAR_invitro	0	2	72	0	1	0	0
CFS	0	0	0	3	0	0	1
IQ	0	16	1	0	677	0	2
MDD	0	1	0	0	0	5	1
OB	0	13	0	1	2	1	134

Table S2. Overlapping number of measured genes.

Phenotype	PA	UC	IAR_invitro	CFS	IQ	MDD	OB
**PA**	19429	17901	18856	17901	17718	17621	17901
**UC**	17901	22836	17933	22836	17713	17935	22836
**IAR_invitro**	18856	17933	19580	17933	17528	17511	17933
**CFS**	17901	22836	17933	22836	17713	17935	22836
**IQ**	17718	17713	17528	17713	18638	17961	17713
**MDD**	17621	17935	17511	17935	17961	19284	17935
**OB**	17901	22836	17933	22836	17713	17935	22836

Table S3: Pre- and post-COCONUT correlation of mean differences for FDR significant genes (FDR < 0.05 and overlapping on all experiments).

Study	Spearman Correlation
GSE16059	0.90
GSE22619	0.88
GSE37146	0.98
MDD(dbGAP)	1
MEXP1425	0.90
GSE20319	0.99
GSE33476	0.89

Table S4: Pre- and post-COCONUT correlation of mean differences for all 16,256 genes.

Study	Spearman Correlation
GSE16059	0.92
GSE22619	0.88
GSE37146	0.94
MDD(dbGAP)	0.90
MEXP1425	0.72
GSE20319	0.90
GSE33476	0.95

Table S5: Absolute Effect Size thresholds for each Phenotype.

Phenotype	Absolute Effect Size Threshold
CFS	0.08
UC	0.40
IAR (in vitro)	77.75
MDD	0.18
OB	44.6
PA	39.9
IQ	0.11
Meta-Phenotype	0.12

Figure S1: Plot of I^2 values versus significance of I^2 (FDR-corrected QEp).

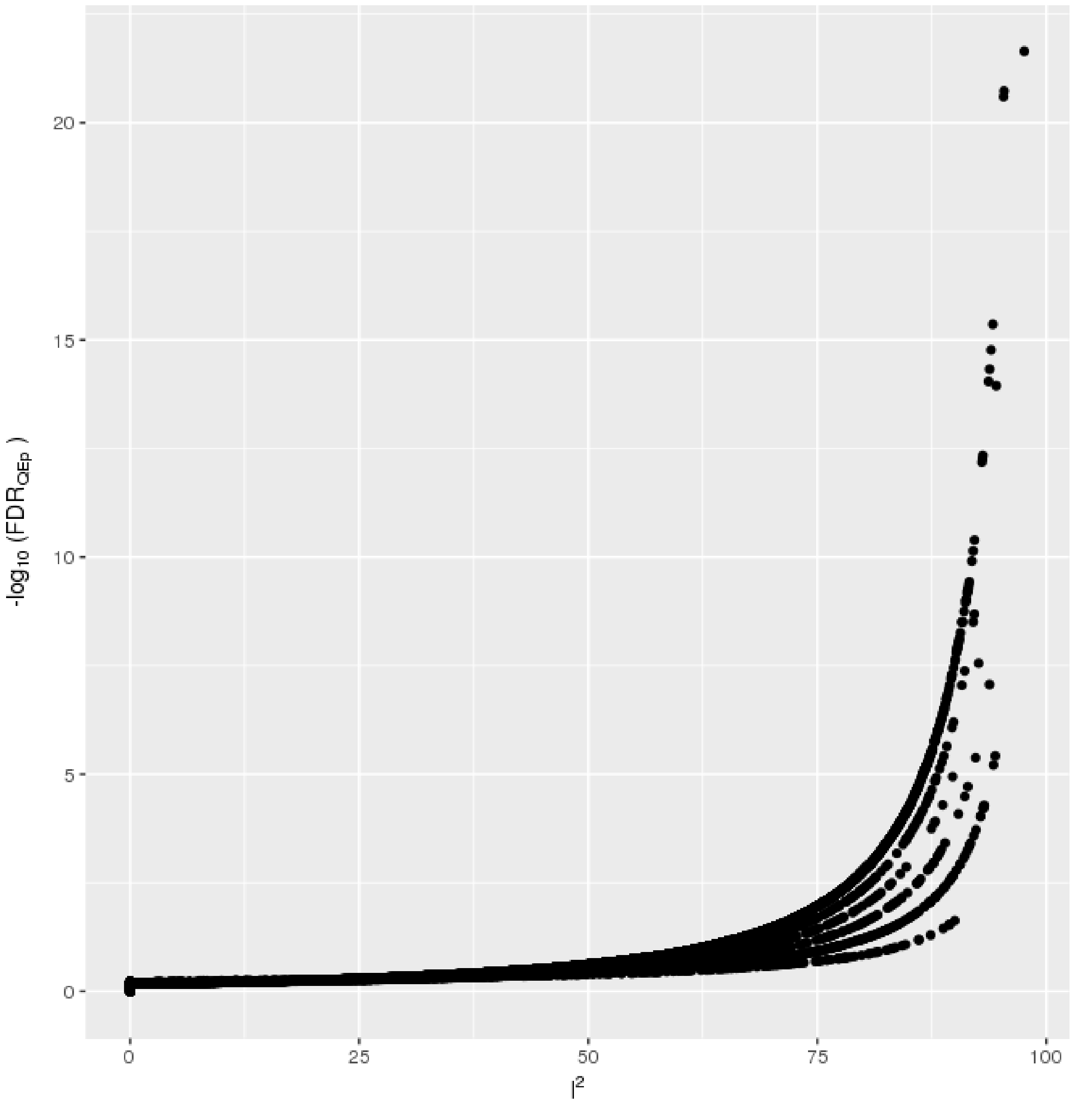

Figure S2: Phenotype-Specific Cumulative Distribution Function (CDF) Plots.

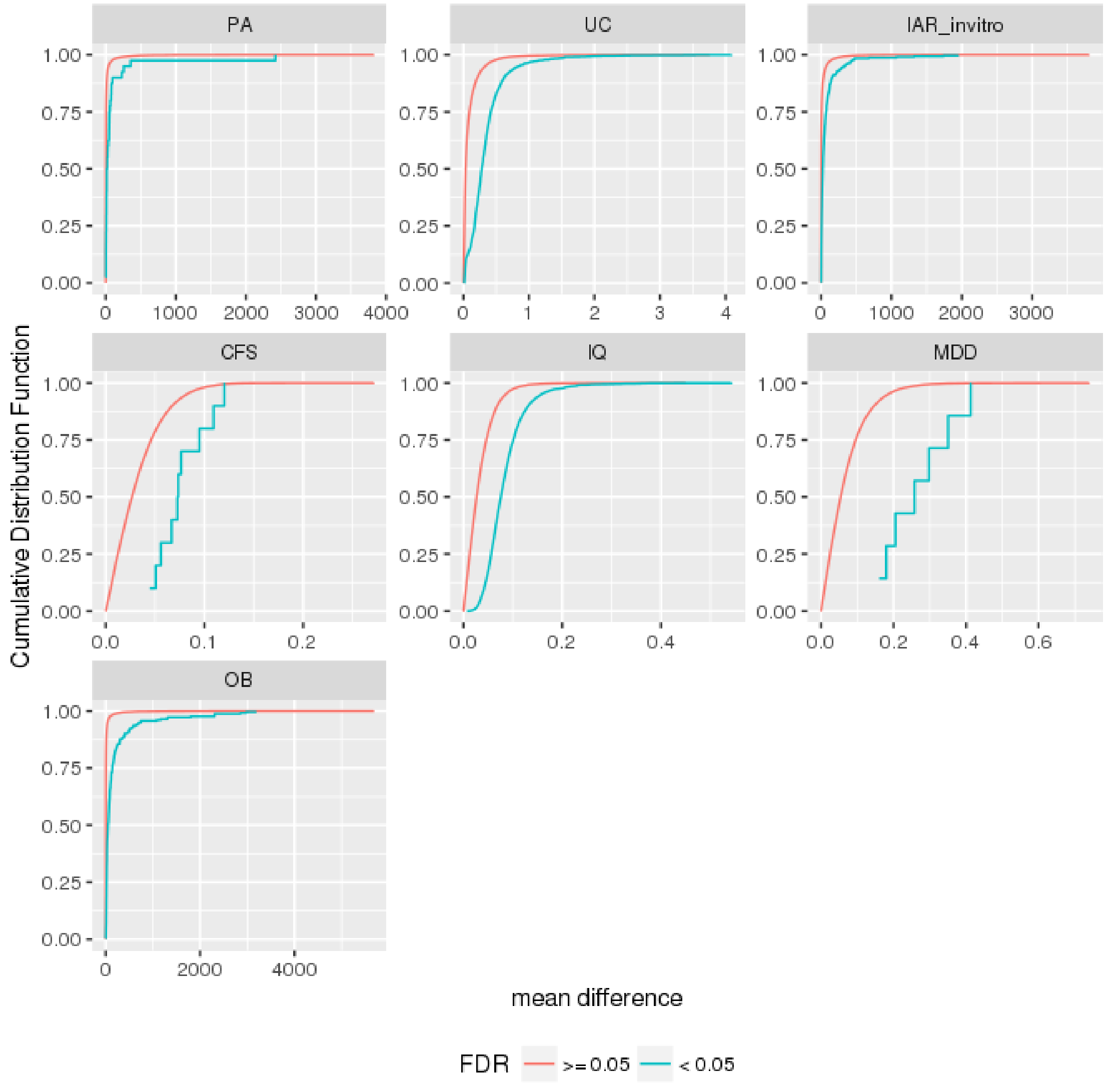

Figure S3: Meta-analytic Cumulative Distribution Function (CDF) Plot.

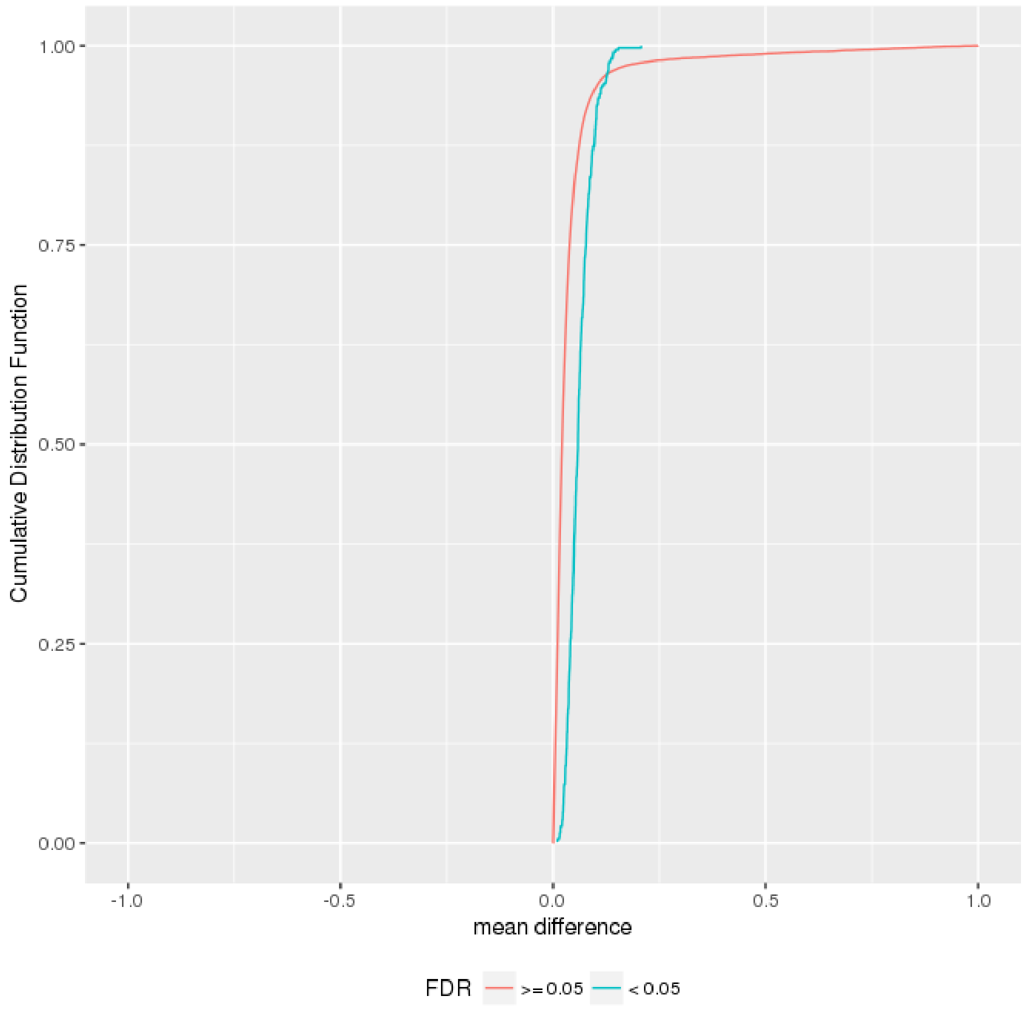

Supplement: Supplementary file 1 — Supplementary Information [file 41598_2017_18585_MOESM1_ESM.pdf]
